# Promoting influenza vaccination among health professionals in Canada: Results of an online survey on Facebook

**DOI:** 10.14745/ccdr.v52i03a03

**Published:** 2026-03-31

**Authors:** Mylène Tantchou Dipankui, Kieran O’Doherty, Lucie Marisa Bucci, Benjamin Giguère

**Affiliations:** 1 University of Guelph, Department of Psychology/College of Social and Applied Human Sciences, Guelph, ON; 2 Canadian Public Health Association, Ottawa, ON

**Keywords:** influenza, Facebook, vaccination, misinformation, health professionals

## Abstract

**Background:**

Vaccination rates of healthcare workers in Canada against influenza are below the national target of 80%. The objective of this study was to identify the preferred promotional formats and types of information that healthcare professionals would consider when deciding whether to get immunized against influenza. The goals of this survey were to 1) inform the design of a social marketing campaign to help healthcare professionals make informed decisions about influenza vaccination, and 2) inform future education and promotional work by vaccination stakeholders.

**Methods:**

A bilingual survey was implemented online using Facebook ads to recruit healthcare professionals across Canada. The survey consisted of 15 mixed multiple-choice and open-ended questions. Eligibility requirements included being a practising Canadian healthcare professional, such as a medical doctor, nurse, pharmacist, nurse practitioner, midwife, or dentist.

**Results:**

A total of 265 healthcare professionals completed the study, with a majority (51.3%) being nurses and practising in Ontario (32.1%). Infographics were viewed as the promotional format most likely to influence their decision-making (33.6%). Healthcare professionals relied on news from various media outlets and peer-reviewed journals (15.8%) to make their decisions. Finally, respondents indicated that influenza vaccine effectiveness was the most relevant information with respect to their decision-making (80.8%).

**Conclusion:**

Infographics may be an important method for promoting influenza vaccination among healthcare professionals. These visual representations should focus on up-to-date information about influenza vaccine effectiveness to respond to the information needs of healthcare professionals. The next steps will be to design a marketing campaign focused on vaccine effectiveness, using infographics as a promotional format.

## Introduction

Influenza is ranked among the top 10 leading causes of death in Canada ((1)). It is associated with an estimated 12,200 hospitalizations and 3,500 deaths per year ((1)). Vaccination remains the most effective preventive strategy to reduce death and hospitalizations due to influenza-related complications in people at high risk of infection ((2)). The National Advisory Committee on Immunization (NACI) recommends that all healthcare professionals receive an influenza vaccine to minimize influenza transmission to people at high risk, such as older adults and young children ((3)). Influenza vaccination among healthcare professionals is considered an essential component of the standard of care for their patients ((2)) however, most recent available vaccination rates for healthcare professionals vary between 32.7 for hospital staff (2024–2025, Ontario) ((4)) and 20.5% (February 2025, Nova Scotia) ((5), which is below the national target of 80% coverage among healthcare professionals ((6)). Data available on the breakdown of vaccine uptake by health profession are rare. In a national cross-sectional study pooling data from the 2007 to 2014 cycles of the Canadian Community Health Survey ((7)), which has been conducted annually by Statistics Canada since 2007, 50% of healthcare professionals reported receiving a seasonal influenza vaccination in the previous 12 months. Vaccination rates varied by occupation type: 72% of family physicians and general practitioners reported receiving the flu shot in the past year, compared to only 4% of chiropractors, midwives, and practitioners of natural healing. Among other groups, 59% of licensed practical nurses and 50% of pharmacists reported being vaccinated.

Strategies to increase vaccination rates among healthcare professionals have included facilitating workplace support for vaccination by increasing access to vaccines, vaccination mandates, and promotion ((8–10)).

Barriers to influenza vaccination among healthcare professionals include perceptions of low risk of infection, concerns about vaccine safety and effectiveness, and misinformation circulating on social media and online networks ((10)). In fact, social media and online networks can be useful in developing professional networks and for patient care and education ((11,12)). However, social media and online networks also provide a medium for disseminating misinformation ((13)), as there are no measures in place to control the quality of the content shared on such portals ((14)). Following Guess and Lyons ((15)), this study defines misinformation as constituting a claim that contradicts or distorts common understandings of verifiable facts. Such misinformation, when spread on social media, can incite negative emotions toward vaccines and may lead to an increase in vaccine hesitancy ((16–18)).

Given the potential health risks associated with sharing inaccurate or out-of-date information, technology companies themselves have taken a position and changed their policies. For example, since February 2019, Pinterest, Facebook, Instagram and Google have addressed anti-vaccine content on their sites and have acknowledged the detrimental role they have played in spreading misinformation about vaccines ((19)). These companies have attempted to reduce access to misleading information about vaccines and promote accurate educational content from expert organizations ((20–22)).

It is in this vein that the Canadian Public Health Association (CPHA) obtained support, in the form of marketing credits, from Facebook Canada to increase awareness of credible vaccine information on social media. The CPHA and Immunize Canada met with project team researchers, including an expert in social marketing campaigns, to discuss the research project’s design and implementation, emphasizing the importance of clearly identifying a target behaviour and audience. Based on numerous discussions and a review of the literature, CPHA and Immunize Canada determined it would launch two distinct campaigns: one that provided credible information to parents of at least one child between 8 and 15 years of age concerning their need to be immunized with the Human Papillomavirus (HPV) vaccine, and another that provided credible information to healthcare professionals concerning their need to be immunized with the annual seasonal influenza vaccine. The results of the HPV campaign were published in 2023 ((23)). The current article focuses on the influenza survey that was performed to prepare the campaign.

A Facebook survey was conducted with healthcare professionals (e.g., nurses, doctors, pharmacists) to collect baseline information on their preferred promotional formats and types of information they would consider when deciding whether to receive an influenza vaccination. The goals of this survey were to 1) inform the design of a social marketing campaign to help healthcare professionals make informed decisions about influenza vaccination (the results of this study will be used to conduct a post-survey campaign to assess the effectiveness of the intervention in a future study), and 2) inform future education and promotional work by vaccination stakeholders. The research questions for this study were: a) what promotional formats do healthcare professionals prefer to receive information about influenza vaccination? and b) what types of information do healthcare professionals consider in their decision-making process to get immunized against influenza?

## Methods

### Participant recruitment

Recruitment took place over an 11 month period, from March 2021 to February 2022. Participant email addresses were collected for a raffle and follow-up survey after the marketing campaign. Informed consent was collected through the Facebook page and by completing the survey. All responses were deidentified before analysis. The project was approved by the Research Ethics Board of the University of Guelph for compliance with federal guidelines for research involving human participants (REB #24-08-004).

### Measures

The survey consisted of 15 multiple-choice and three open-ended questions, and took approximately 15 minutes to complete (see survey questions in the **Appendix**, **Supplemental A**). SimpleSurvey was used as the survey platform, with randomized questions. The following topics were covered:

1) Sociodemographic information (e.g., age, province/territory where their practice is located, sex, gender, job title)

2) Influenza vaccine decision-making process

3) Influenza information sources

4) The advertising formats most likely to influence the decision-making

5) Trust in public health messages

This article focuses on topics 3 and 4, as the answers for these topics were required to implement the marketing campaign. Items specific to social media sources, preferred promotional formats, and the type of information healthcare professionals would consider in their decision-making process (questions 3 and 4) were developed in collaboration with Immunize Canada, a national coalition of non-governmental, professional, health, government, and private sector organizations dedicated to promoting the benefits of immunization and questions ((24)). At the end of the questionnaire, participants were invited to provide their email addresses to be recontacted for the post-campaign survey.

### Mitigation of online surveying issues

Online recruitment for surveys can present challenges, including fraudulent participants, low recruitment rates, incomplete responses, and high attrition. To address low recruitment, the recruitment period was expanded. To identify fraudulent participants, participant lists were reviewed by two team members to identify fraudulent email addresses (e.g., ones that used inappropriate words or those bounced when trying to recontact the participant), which were discarded. Respondents who did not complete at least 80% of the survey were also removed. To reduce attrition, team members sent two reminders to non-responders to optimize the response rate. Finally, survey responses were monitored while the survey was still live, and the number of participants was updated during team meetings.

### Data analysis

Survey responses were analyzed using a deductive and inductive approach. To select variables, the data set was reviewed to identify the variables. Once the key questions necessary to launch the marketing campaign were identified, the team discussed the variables that might be relevant to answer the research questions and reached a consensus on which to include in the research. A threshold of 20% or more non-completion rate was adopted. Variables were checked to identify univariate outliers. The responses to questions about influenza information sources were grouped into two main variables: 1) social media sources, and 2) “other” sources. Social media sources combined the responses of participants who stated that they relied on Facebook, Twitter, Instagram, Pinterest, and/or Reddit to obtain credible information about vaccines and vaccination for influenza. Other sources combined the responses of participants who relied on scholarly and/or non-scholarly sources that were not considered social media to obtain credible information about vaccines and vaccination for influenza (see **Table 1** and **Table 2** for more details).

**Table 1:** Frequency table of social media sources that survey participants rely on to obtain credible information about influenza vaccines and vaccination

Social media source	Participants ranking social media first	Percent (%)	Valid percent (%)
Facebook	77	29.1	38.1
Twitter	17	6.4	8.4
Instagram	11	4.2	5.4
Pinterest	7	2.6	3.5
Reddit	15	5.7	7.4
Other	75	28.3	37.1
Total	202	76.2	100.0
Missing	63	23.8	N/A
Total	265	100.0	N/A

Abbreviation: N/A, not applicable

**Table 2:** Frequency table of “other” sources that survey participants rely on to obtain credible information about influenza vaccination

Sources	N	Percent (%)
Non scholarly sources	64	65.3
Scholarly sources	34	34.7
Total	98	100.0

## Results

### Participant demographics

A total of 524 healthcare professionals participated in the study. A total of 259 were excluded because up to 80% of their forms were not completed. Finally, 265 healthcare professionals completed the survey, with a majority (51.3%) of them being nurses. A total of 83.4% of respondents were female and 50.6% were in the range of 25–44 years old. Most of the participants practiced in Ontario (32.1%) and Québec (19.6%), and only a few were practicing in the territories (Yukon [n=1; 0.4%] and Nunavut [n=1; 0.4%]). These results are summarized in **Table 3**.

**Table 3:** Frequency table of the demographic characteristics of survey participants^a^

Demographics characteristics	Number of respondents (n)	Percent (%)
**Age (years)**	**248**	**93.6**
18–24	13	4.9
25–34	67	25.3
35–44	67	25.3
45–54	51	19.2
55–64	44	16.6
65–74	6	2.3
Missing	17	6.4
Total	265	100.0
**Sex**	**248**	**93.6**
Female	221	83.4
Male	24	9.1
Choose not to respond	3	1.1
Missing	17	6.4
Total	265	100.0
**Gender**	**248**	**93.6**
Woman	220	83.0
Man	25	9.4
Other	3	1.1
Missing	17	6.4
Total	265	100.0
**Province of residence**	**247**	**93.2**
Alberta	30	11.3
British Columbia	32	12.1
Manitoba	16	6.0
New Brunswick	7	2.6
Newfoundland and Labrador	2	0.8
Nova Scotia	13	4.9
Nunavut	1	0.4
Ontario	85	32.1
Québec	52	19.6
Saskatchewan	8	3.0
Yukon	1	0.4
Subtotal	247	93.2
Missing	18	6.8
Total	265	100.0
**Job title**	**248**	**93.6**
Medical doctor (MD)	17	6.4
Nurse	136	51.3
Pharmacist	10	3.8
Dentist	4	1.5
Other	81	30.6
Missing	17	6.4
Total	265	100.0

^a^ A total of 265 (100%) healthcare professionals completed the survey. Of these, 247 (93.2%) answered the question related to their location, and 18 did not provide a response (missing data: 6.8%, n=18), leading to a total of 100%

Social media sources that participants rely on to obtain credible information about vaccines and vaccination for influenza

The survey was conducted on Facebook. This platform was the primary social media source that most surveyed healthcare professionals reported relying on to find credible information about influenza vaccines and vaccination (29.1%; n=77) (See Table 1). “Other” was selected as the second most relevant source (28.3%; n=75). Twitter is the third most relevant social media source (6.4%; n=17).

Promotional formats used to provide influenza vaccination information that may influence healthcare professionals’ decision-making

Participants were asked to rank a series of promotional formats used to provide information about vaccination for influenza in order of most likely “1” to least likely “5” to influence their decision-making, ensuring that no two formats received the same score (i.e., two promotional formats could not both be scored the same). The data was then combined to identify the promotional format that each participant ranked first. Infographics were the most frequently selected format, with 89 participants ranking them first as the influential. “Other” sources (e.g., scientific papers), were ranked second (total of 42), “videos” were ranked third (total of 34), and “narratives/stories” were ranked fourth (total of 21) (see **Table 4**).

**Table 4:** Frequency table of promotional formats used to provide influenza vaccination information from most likely “1” to least likely “5” to influence decision-making

Promotional formats	Participants ranking the format first	Percent (%)	Valid percent (%)
Videos	34	12.8	17.3
Images	11	4.2	5.6
Infographics	89	33.6	45.2
Narratives/stories	21	7.9	10.7
Others	42	15.8	21.3
Total	197	74.3	100.0
Missing	68	25.7	N/A
Total	265	100.0	N/A

Abbreviation: N/A, not applicable

Other sources identified by participants as potentially influencing their decision-making about influenza vaccines and vaccinations were grouped into two main categories: scholarly sources and non-scholarly sources. Scholarly sources included peer-reviewed journals, reports and the websites of government agencies. Non-scholarly sources included social media, audio and video messages, and personal experiences. The majority of participants (65.3%, n=64) relied on non-scholarly sources, while 34.5% (n=34) relied on scholarly sources (see Table 2). Among non-scholarly sources, healthcare professionals relied mainly on news from various media, such as audio broadcasts, videos and press releases (20.3%; n=13). They also relied on information from their workplace (14.1%; n=9). Finally, 6.3% of healthcare professionals (n=4) reported that promotion materials do not influence their decision-making. These results are summarized in **Table 5**. The themes and categories are presented in the supplemental material.

**Table 5:** Frequency table of non-scholarly sources the participants rely on to obtain credible information about influenza vaccination

Non-scholarly sources	N	Percent (%)
News	13	20.3
Personal experience	3	4.7
Information from the healthcare system	9	14.1
Posts on social media	2	3.1
Ad non-influential	4	6.3
Other, non-classifiable sources	10	15.6
Don’t know	11	17.2
None	12	18.8
Total	64	100.0

### Information that healthcare professionals consider when making decisions about influenza vaccination

The most frequently sought information by healthcare professionals when deciding whether to receive an influenza vaccine was vaccine effectiveness (n=214), followed by clinical trial data (n=156). The potential side effects of vaccines were the third most frequent option selected (n=148). Who monitors vaccine safety (n=107) and who regulates vaccine safety (n=107) were also ranked as important. How to minimize vaccine injection pain and fear did not have a high ranking (total of 19), and neither did the alternative influenza vaccination schedule (total of 18). These results are summarized in **Table 6**.

**Table 6:** Information that healthcare professionals consider when making their decisions about vaccination for influenza^a,b^

Information healthcare professionals look for to make their decisions about vaccination for influenza	Number of respondents (n)	Percent (%)
Effectiveness of vaccines	214	80.8
Clinical trial data	156	58.9
Potential side effects of vaccines	148	55.8
Who monitors vaccine safety	107	40.4
Who regulates vaccine safety	107	40.4
How vaccines are tested	86	32.5
Vaccine ingredients	71	26.8
How to minimize vaccine injection pain and fear	19	7.2
Alternative vaccination schedules	18	6.8
Don’t know/Not sure	11	4.2
Homeopathic remedies	2	0.8
Total	265	100.0

^a^ Respondents could select multiple responses

^b^ Mean=1.00; Median=1.00

## Discussion

The objective of this study was to explore the promotional formats and types of information that healthcare professionals consider when making their decision about influenza vaccines and vaccination. The study found that healthcare professionals surveyed considered infographics the most influential format for supporting their decision-making about regarding influenza vaccines and vaccination. They also identified the vaccine effectiveness as the most relevant information for this process.

An infographic is a visual representation of educational content ((25)). Infographics are commonly used to share complex information, communicate scientific facts, and drive behavioural change ((25)). When infographics are designed properly, they can engage both specialists and lay persons ((26)). Li *et al*. suggest that an infographic that is properly designed can illustrate concepts, clarify data patterns, and provide aesthetic pleasure. Visually simple infographics—in which the visual message presents orderliness, balance, and clarity—may be more effective in driving behavioural change compared to ones that are complex ((26)).

Even if a vaccination campaign focusing on educating and promoting influenza vaccination may be sufficient to change some of the underlying misconceptions about influenza and vaccine use, information alone will not result in significant changes in behaviour ((27)). In a recent study aiming to analyze strategies to increase vaccination coverage in healthcare workers, Schumacher *et al.* ((28)) noted that vaccination campaigns focusing on education and promotion of influenza vaccination only increased vaccine coverage from 25% to 40% in studies with low initial vaccine coverage. They also noted that education and promotional aspects were used as the basis of all campaigns, but when implemented as the sole key intervention, absolute vaccine coverage did not exceed 40%. These authors suggest that campaigns based on education and promotion, or on-site-vaccination, should be combined with other approaches, such as vaccination stands and incentives to achieve high overall vaccination coverage.

Effectiveness of influenza vaccines is the main information that healthcare professionals seek when making their decisions about influenza vaccines and vaccination. Each year, researchers conduct studies to determine how well influenza vaccines work to protect against influenza. However, estimates of how well an influenza vaccine works vary based on study design, outcome(s) measured, population studied, and type of influenza vaccine ((29)). Influenza vaccine effectiveness primarily depends on the vaccinated individual’s characteristics, such as age and health status, and how closely the vaccine matches the prevalent virus strains circulating in the community ((29)). It can also depend on the infection history of an individual ((30)). Therefore, influenza vaccine effectiveness can vary from season to season. In Canada, NACI provides annual recommendations regarding the use of authorized influenza vaccines to the Public Health Agency of Canada ((31)). These recommendations are based on up-to-date information on influenza epidemiology, immunization practices and influenza vaccine products authorized and available for use in Canada. The outcomes of the survey from the current study support the importance of sharing this up-to-date information about influenza vaccine effectiveness and associated recommendations with healthcare professionals. However, healthcare professionals must be vaccinated at the start of influenza season, when relevant vaccine effectiveness estimates are not yet available and typically become known only after the influenza season has ended. Therefore, it might be relevant to share general influenza vaccine effectiveness statistics, including those from the previous year, with healthcare professionals. The outcomes of the survey also suggest that the use of the infographics may be important in communicating this information.

This study demonstrated a number of strengths. As far as is known, this study is the first conducted in Canada to identify the preferred format and type of information that healthcare professionals will consider when deciding whether to receive an influenza vaccination. As such, the main strength of this study is to contribute to increase the body of knowledge on strategies to increase influenza vaccination uptake among healthcare professionals using Facebook. Since healthcare professionals across the country participated in the study, the diversity of the sample makes the results applicable to various healthcare settings. The study’s focus on the promotional formats and types of information that healthcare professionals are looking when deciding on influenza vaccination adds practical significance to its results, as it could direct the decisions related to the goal of improving influenza vaccination among healthcare professionals.

### Limitations

The study was limited in several ways. Since the study was underway at the start of COVID-19, participant responses may have been influenced by “survey fatigue” ((32)) due to their involvement in multiple studies. This shortcoming was mitigated by allowing participants to complete the study at their own pace and within a reasonable time.

Facebook surveys themselves have reliability issues, such as incidences of fraudulent participants and the use false emails by participants, out of concern that their information may be distributed to third parties and misused ((33)). Validating participant identities by matching IP addresses to reported provinces or territories would have been helpful to identify more false emails.

On Facebook, the users’ opinions are easily accessible to other users. This may have led participants to being influenced by the opinions of other people. Also, participants may have responded with what they believe researchers wanted to hear, rather than honestly expressing their own thoughts ((33)).

In addition to a small sample of a social media platform users, most respondents reported being female and nurses located in Ontario or Québec, which is not representative of the entire breadth of healthcare professionals in Canada. Therefore, the limited representation of many sectors of the health profession makes it difficult to generalize the study’s results to all healthcare professionals.

The percentage of missing data could affect the generalizability of the results. This bias was mitigated by reporting both the percentage and valid percentage in the tables to reflect the actual data.

Finally, one limitation that arose as part of the work is the challenge of reliably coding the preferred media type used in communication. Future research could include developing a focused coding process that aims to address the issue of preferential communication in interventions ((34)).

## Conclusion

The results of this study suggest that infographics may be an important method for promoting influenza vaccines and vaccination among healthcare professionals on Facebook. These visual representations should focus on vaccine effectiveness to respond to the information needs of healthcare professionals. However, because influenza vaccine effectiveness can vary from season to season, and this information is only available after the influenza season, it appears important to share up-to-date information or general statistics about influenza vaccines effectiveness and recommendations with healthcare professionals when designing infographics. The next steps will be to design a marketing campaign focused on vaccine effectiveness, using infographics as a promotional format. Following the marketing campaign, it will be possible to measure healthcare professionals’ knowledge, attitudes and perceptions of influenza vaccine and vaccination, allowing for an assessment of the social marketing campaign’s effectiveness in using infographics as a communication tool.

## Appendix

Supplemental material is available upon request to the author: mtantcho@uoguelph.ca

Supplemental A: Facebook Survey with Health Professionals on Influenza Vaccination
